# A Novel Modified Bu/Vp16/cy/Flu/Ara‐C Conditioning Regimen Enhances Outcomes for High‐Risk Acute Lymphoblastic Leukemia Patients Undergoing Allogeneic Hematopoietic Stem Cell Transplantation

**DOI:** 10.1002/cam4.71669

**Published:** 2026-02-28

**Authors:** Xiaoyan Zhao, Yifan Yao, Yan Sun, Ziwei Xu, Aiguo Liu, Xin Dong, Huafang Wang

**Affiliations:** ^1^ Institute of Pediatrics, Tongji Hospital, Tongji Medical College Huazhong University of Science and Technology Wuhan China; ^2^ Institute of Hematology, Union Hospital, Tongji Medical College Huazhong University of Science and Technology Wuhan China; ^3^ Institute of Thoracic Surgery, Union Hospital, Tongji Medical College Huazhong University of Science and Technology Wuhan China

**Keywords:** acute lymphoblastic leukemia, conditioning regimen, relapse, transplantation

## Abstract

**Purpose:**

Allogeneic hematopoietic stem cell transplantation (allo‐HSCT) effectively treats high‐risk acute lymphoblastic leukemia (ALL), yet challenges persist due to post‐transplant relapse and conditioning regimen toxicities. The determination of an appropriate preconditioning regimen is critical to improving patient outcomes. In our transplant center, we have modified the traditional busulfan (Bu)/cyclophosphamide (Cy)/etoposide (Vp16) protocol by adding fludarabine (Flu) and cytarabine (Ara‐C), while reducing the dosage of Cy. This novel modification seeks to enhance transplantation outcomes for ALL patients.

**Methods:**

This study retrospectively collected clinical data from 88 high‐risk ALL patients who received transplantation from June 2018 to December 2023. Among these patients, 40 received the novel modified Bu/Cy/Vp16/Flu/Ara‐C conditioning protocol, while 48 received the traditional Bu/Cy/Vp16 regimen and served as a control group.

**Results:**

This study demonstrated a notably reduced incidence of cardiac toxicity in patients treated with the modified Bu/Cy/Vp16/Flu/Ara‐C conditioning compared to those on the traditional Bu/Cy/Vp16 regimen. Furthermore, other types of conditioning‐related toxicities were within acceptable limits in the modified regimen group. Regarding efficacy, the Bu/Cy/Vp16/Flu/Ara‐C protocol significantly reduced the cumulative two‐year relapse rate in high‐risk ALL patients compared to the Bu/Cy/Vp16 scheme (38.7% (20.9%–52.5%) vs. 11.8% (0.2%–22.1%), *p* = 0.017). The modified regimen showed significant improvements in 2‐year overall survival at 71.6% (57.1%–89.6%) compared to 50.6% (38%–67.3%) (*p* = 0.048), and in two‐year disease‐free survival at 66.7% (51.9%–85.6%) compared to 45.3% (33.1%–62.1%) (*p* = 0.015). Transplant‐related mortality was comparable between the two groups. A subgroup analysis based on disease status (CR1 and ≥ CR2) revealed that high‐risk ALL patients on the modified regimen had lower relapse rates and significantly better OS and DFS than those on the Bu/Cy/Vp16 scheme.

**Conclusions:**

The novel modified Bu/Cy/Vp16/Flu/Ara‐C conditioning significantly enhances the prognosis of high‐risk ALL patients receiving transplantation, especially those in CR1.

## 1 Introduction

Acute lymphoblastic leukemia (ALL) is an aggressive blood cancer marked by the abnormal proliferation of immature lymphocytes, comprising about 20% of adult leukemia cases [1]. Allogeneic hematopoietic stem cell transplantation (allo‐HSCT) is the main curative treatment for high‐risk, relapsed, or refractory ALL [2, 3]. The conditioning regimen is one of the most critical factors determining outcomes for ALL patients receiving allo‐HSCT [4, 5, 6]. However, the optimal preparative regimen for these patients remains undetermined.

In traditional preparative protocols, total body irradiation (TBI)‐based protocols, while demonstrating clear advantages in eradicating leukemic cells and reducing relapse risk [7, 8], are limited in their clinical application due to long‐term toxicities and short‐term complications [9]. Consequently, non‐TBI regimens based on busulfan (Bu), such as the classic Bu‐cyclophosphamide (Bu‐Cy) regimen, have also been developed [10, 11]. However, current evidence remains controversial regarding whether TBI‐based or Bu‐based regimens provide superior treatment efficacy. One study indicates no significant difference in outcomes between Bu‐Cy and TBI‐Cy regimens for patients with B‐ALL, with 2‐year overall survival (OS) rates of 76.6% versus 79.4%, and 2‐year relapse rates of 20.2% versus 18.4%, respectively [10]. In contrast, other studies suggest that the anti‐leukemic potency of the Bu‐Cy regimen may be less effective than that of TBI‐based regimens. One study in ALL patients (≤ 35) reported a 5‐year leukemia‐free survival rate of only 18% in the Bu‐based group, significantly lower than the 50% in the TBI group [8]. Another large‐scale study also showed that the 3‐year cumulative relapse rate was 37% in the Bu group, compared to 28% in the TBI group (*p* = 0.007) [12].

Recently, researchers have begun exploring the incorporation of drug combinations with synergistic effects into Bu‐based regimens. Among these, the combination of fludarabine (Flu) and cytarabine (Ara‐C) shows promising potential. The key mechanism of action is that administering Flu prior to Ara‐C inhibits ribonucleotide reductase, reduces intracellular levels of deoxycytidine triphosphate (dCTP), thereby promoting the phosphorylation of Ara‐C into its active form Ara‐CTP and enhancing its incorporation into DNA, ultimately resulting in a synergistic anti‐leukemic effect [13, 14]. This synergistic effect has been preliminarily validated in acute myeloid leukemia: compared to the Bu/Cy2 regimen, patients receiving the Flu, Ara‐C, and Bu combination showed a significantly improved 3‐year GVHD‐free, relapse‐free survival (31.20% vs. 14.96%) [15]. In recent years, this strategy has begun to gain attention in ALL as well. One study combined Flu, Ara‐C, Bu, Cy, anti‐thymocyte globulin (ATG), and post‐transplant Cy (PT‐Cy) to create an intensified conditioning regimen, aiming to enhance disease control through the Flu/Ara‐C synergy. The results indicated a trend towards improved 2‐year graft‐versus‐Host Disease (GVHD)‐free, relapse‐free survival in pediatric patients with high‐risk AML and ALL receiving this PTCy‐based intensified regimen (63.3%) compared to the modified Bu‐Cy plus ATG control group (35.4%) (*p* = 0.092) [16], suggesting its potential for reducing relapse. However, its definitive anti‐leukemia efficacy in adult ALL requires further confirmation through additional studies. Besides, in terms of safety, the administration of high‐dose Cy in Preparative protocols constitutes a well‐defined dose‐limiting toxicity source, with its cardiotoxicity being dose‐dependent [17]. Notably, even within the conventional clinical dose range (120 or 100 mg/kg), Cy‐related cardiac adverse events remain a significant concern [18], further limiting its safe application in allo‐HSCT conditioning.

Given these considerations, our study aims to optimize the traditional Bu/Vp16/Cy regimen through two key modifications: first, introducing the Flu and Ara‐C combination to leverage their proven synergistic anti‐leukemic activity for enhanced myeloablative effect; second, correspondingly reducing the Cy dose, aiming to retain its necessary immunomodulatory function while mitigating toxicities. To this end, we performed an investigation to systematically assess the efficacy and safety of the modified Bu/Vp16/reduced‐dose‐Cy/Flu/Ara‐C regimen compared to the conventional Bu/Vp16/standard‐dose‐Cy protocol in allo‐HSCT for high‐risk ALL patients, hoping to provide new evidence‐based insights for optimizing transplant conditioning strategies.

## 2 Methods

### 2.1 Eligibility Criteria

This study enrolled 88 high‐risk ALL patients underwent allo‐HSCT at our center between June 2018 and December 2023. Inclusion criteria: this study enrolled patients aged 18–60 years with high‐risk ALL who voluntarily received allo‐HSCT. High‐risk ALL was defined as meeting any of the following criteria: (1) being in the second or subsequent complete remission (≥ CR2); or (2) being in first CR1 with at least one of the following high‐risk factors: hyperleukocytosis at initial diagnosis (B‐ALL > 30 × 10^9^/L, T‐ALL > 100 × 10^9^/L), age > 35 years, positive minimal residual disease (MRD), or the presence of adverse cytogenetic abnormalities (e.g., Ph^+^, t(4;11), complex karyotype, low hypodiploidy, etc.); delayed CR1 (> 28 days of induction therapy) [19, 20, 21]. Exclusion criteria included: uncontrolled active infection (viral, bacterial, or fungal), concomitant severe underlying cardiac, hepatic, pulmonary, or renal disease, or a prior history of other systemic malignancies. The conditioning regimen was assigned based on the enrollment period: 48 patients enrolled between June 2018 and May 2020 received the traditional Bu/Cy/Vp16 regimen, while 40 enrolled between June 2020 and December 2023 received a modified regimen that combined Flu and Ara‐C with the traditional protocol (Bu/Cy/Vp16/Flu/Ara‐C). The follow‐up deadline for the study was December 1, 2024. Patients were divided into two cohorts according to their conditioning regimens: the ‘Bu/Vp16/Cy group’ for those receiving Bu/Vp16/Cy, and the ‘Bu/Vp16/Cy/Flu/Ara‐C group’ for those receiving Bu/Vp16/Cy/Flu/Ara‐C. The Ethics Committee of Union Hospital, Huazhong University of Science and Technology approved this study.

### 2.2 Conditioning Regimens

ALL patients receiving allo‐HSCT were administered one of two myeloablative preparative regimens: Bu/Vp16/Cy or Bu/Vp16/Cy/Flu/Ara‐C. The Bu/Vp16/Cy regimen consisted of administering intravenous Bu at 3.2 mg/kg/day from Day −9 to day −7, followed by Vp16 at 5 mg/kg every 12 h from Day −6 to Day −5, and concluding with Cy at 60 mg/kg/day from Day −4 to Day −3. The Bu/Vp16/Cy/Flu/Ara‐C regimen included intravenous Flu (30 mg/m^2^/day) from Day −10 to −7, alongside Vp16 (5 mg/kg every 12 h) on Days −10 and −9. Intravenous Bu (3.2 mg/kg/day) was administered from Day −8 to −6, while Ara‐C (1 g/m^2^/day) was given from Day −8 to −7, with infusions starting 2 h post‐Flu administration. Finally, Cy (1.0 g/m^2^/day intravenously) was infused from Day −5 to −4.

### 2.3 Stem Cell Source

Hematopoietic stem cells were mobilized in donors through daily subcutaneous injections of granulocyte colony‐stimulating factor at 8–10 μg/kg. The collected unmanipulated stem cells were directly infused into ALL recipients. Specifically, for 5/10 HLA‐matched haploidentical transplants, patients received combined grafts of bone marrow‐derived and peripheral blood (PB)‐derived stem cells. For all other allo‐HSCT procedures, exclusively PB‐derived stem cells were infused.

### 2.4 Prevention of GVHD

All patients experienced prophylactic protocols tailored to the type of transplant. For HLA‐matched sibling transplants, patients were administered cyclosporine A (CsA) at 5 mg/kg twice daily from Day −1, alongside methotrexate (MTX) at 15 mg/m^2^ on Day +1 and 10 mg/m^2^ on Days +3, +6. For unrelated or haploidentical transplants, the regimen included basiliximab at 20 mg intravenously on Days 0 and +4, along with mycophenolate mofetil at 0.5 g orally twice daily, in addition to the CsA/MTX regimen (MTX at 15 mg/m^2^ on Day +1 and 10 mg/m^2^ on days +3, +6 and +11). The +11 day dose of MTX may be omitted in cases of severe oral mucositis. The strategy for tapering immunosuppressants following transplantation is as follows [22]: for MMF in the setting of unrelated donor or haploidentical HSCT, the dose is typically halved once engraftment is achieved and then discontinued at 2–3 months post‐transplant. For CsA, in matched sibling donor transplantation, tapering is typically initiated at 3 months post‐transplant and discontinued after +6 months; for haploidentical and unrelated donor transplantations, CsA tapering usually begins around day +100, with therapy typically discontinued between 6 and 9 months after transplantation. In actual clinical practice, the regimen should be individually adjusted according to the patient's relapse risk, disease status, infections and occurrence of GVHD, with the therapy duration either shortened or extended as appropriate. In haploidentical transplants at our center, ATG was administrated at 3 mg/kg (Days −1 to 0) for donor‐recipient pairs with over 5/10 HLA‐matched loci, and for 3 days (Days −3 to −1) when exactly 5/10 loci were matched.

### 2.5 Supportive Care

To prevent epilepsy, the patients took oral phenobarbital 100 mg three times daily from the beginning to the end of the conditioning protocol. Systemic infection prophylaxis includes prevention of bacterial, fungal, and viral infections. Specifically, from Day −10 to Day −2, the patients took valganciclovir at 5 mg/kg twice daily. Additionally, moxifloxacin 400 mg per day was administered orally every day, and fluconazole was administered from Day −10 until engraftment. For viral monitoring, Epstein–Barr Virus and Cytomegalovirus DNA will be tested weekly during the first 100 days post‐transplantation, biweekly until 6 months, and monthly thereafter until 1 year post‐transplant. If CMV reactivation occurs, preemptive treatment with ganciclovir or foscarnet would be initiated. In addition, disease status assessments, including bone marrow cytology and MRD testing, will be conducted monthly within the first 6 months post‐transplant. This study employed a flow cytometry–based MRD monitoring approach with a sensitivity threshold of 10^−4^. After that, until 24 months post‐transplant, comprehensive evaluations will be performed every 3 months.

### 2.6 Engraftment, Chimerism and Regimen‐Related Toxicities Assessment

Neutrophil engraftment is defined as achieving an absolute neutrophil count of ≥ 0.5 × 10^9^/L for three consecutive days, and a platelet count exceeds 20 × 10^9^/L without transfusion for the subsequent 7 days, signifying successful platelet engraftment. Chimerism was evaluated using fluorescence in situ hybridization or quantitative polymerase chain reaction. Regimen‐related toxicities (RRTs) were evaluated in accordance with Bearman's Grading System [23].

### 2.7 Statistical Analysis

The study's data analysis utilized SPSS 26.0, GraphPad Prism 9.5, and R 4.5.0. The Mann–Whitney *U* test was employed for continuous variables, and the Chi‐square or Fisher's exact test was used for categorical data. OS was defined as the time from transplantation to death from any cause or the date of last follow‐up [10]. Disease‐free survival (DFS) was defined as the time from transplantation to relapse, death from any cause, or the date of last follow‐up [10]. Transplant‐related mortality (TRM) was defined as the time from transplantation to death related to transplantation [24]. Relapse was defined as meeting any of the following criteria [25]: hematologic relapse refers to the detection of leukemia cells in the peripheral blood, or a proportion of bone marrow blasts ≥ 5%, or the occurrence of extramedullary leukemia cell infiltration in patients have achieved CR after transplantation; cytogenetic relapse refers to the reappearance of the original cytogenetic abnormalities, or the emergence of a certain proportion of recipient chimerism in sex chromosomes from a complete donor type, in patients who have achieved complete cytogenetic remission after transplantation; molecular relapse is defined as the detection of specific or non‐specific molecular biomarker abnormalities by flow cytometry and/or polymerase chain reaction that exceed predefined thresholds, yet do not meet the criteria for hematological relapse. To estimate the cumulative rate of TRM, relapse, GVHD, a competing risk model was utilized, and comparisons were made using the Gray's test. For the analysis of GVHD, relapse, and TRM, the competing events were referred to as death without GVHD, death without relapse, and relapse, respectively. Kaplan–Meier method was employed to analyze OS and DFS, with statistical comparisons conducted via the Log‐rank test. In the multivariate analysis, factors influencing OS and DFS were assessed using the Cox proportional hazards regression model, while factors influencing relapse were analyzed using a competing risks regression model, with the competing event for relapse defined as death without relapse. Univariate analysis included the following variables: age, gender, conditioning regimen, pre‐transplant MRD status, occurrence of aGVHD, and occurrence of cGVHD. Variables with a *p*‐value < 0.10 in the univariate Cox analysis, along with variables known to have significant impacts on prognosis, were included in the multivariate Cox analysis. The final variables incorporated into the multivariate Cox regression model were the following four: conditioning regimen, pre‐transplant MRD status, occurrence of aGVHD, and occurrence of cGVHD. The *p*‐values were two‐sided, with significance set at *p* < 0.05.

## 3 Results

### 3.1 Patient Characteristics

Table 1 indicates no remarkable differences in baseline clinical characteristics between high‐risk ALL patients in the Bu/Vp16/Cy/Flu/Ara‐C group and those in the Bu/Vp16/Cy cohort.

**TABLE 1:** The characteristics of ALL patients underwent allo‐HSCT.

	Bu/Vp16/Cy (*n* = 48)	Bu/Vp16/Cy/Flu/Ara‐C (*n* = 40)	*p*
Patient median age, years (range)	25 (11–51)	32 (13–57)	0.108
Gender			0.582
Male	26 (54.2)	24 (60.0)	
Female	22 (45.8)	16 (40.0)	
Subtype			0.333
T‐ALL	16 (33.3)	8 (20.0)	
B‐ALL	30 (62.5)	31 (77.5)	
Unspecified	2 (4.2)	1 (2.5)	
Molecular subtype			
BCR::ABL1	11 (22.9)	10 (25.0)	0.819
MLL	4 (8.3)	5 (12.5)	0.773
PAX5alt	5 (10.4)	5 (12.5)	1.000
TCF3::PBX1	5 (10.4)	2 (5.0)	0.590
IKZF1	2 (4.2)	1 (2.5)	1.000
Low hypodiploid	3 (6.3)	3 (7.5)	1.000
Pre‐transplant MRD			0.850
Positive	10 (20.8)	9 (22.5)	
Negative	38 (79.2)	31 (77.5)	
Disease status			0.899
CR1	33 (68.8)	28 (70.0)	
≥ CR2	15 (31.3)	12 (30.0)	
Pre‐transplant cyclophosphamide	14 (29.2)	10 (25.0)	0.662
Donor‐recepient gender			0.258
Mismatched	25 (52.1)	16 (40.0)	
Matched	23 (47.9)	24 (60.0)	
Donor‐recipient HLA matching			0.850
Donor/recipient‐related HSCT	38 (79.2)	31 (77.5)	
HLA matching: 10/10	9 (18.8)	8 (20.0)	
HLA matching: 9/10	4 (8.3)	3 (7.5)	
HLA matching: 5/10 to 8/10	25 (52.1)	20 (50.0)	
Donor/recipient‐unrelated matched HSCT	10 (20.8)	9 (22.5)	
ABO match			0.937
Mismatched	28 (58.3)	23 (57.5)	
Matched	20 (41.7)	17 (42.5)	
Median CD34+ cells, ×10^6^/kg (range)	5.96 (2.12–21.09)	7.37 (2.57–15.53)	0.127
Median nucleated cells, ×10^8^/kg (range)	14.08 (5.63–24.25)	12.45 (5.96–29.11)	0.497
Median time of diagnosis to transplant, days (range)	143.5 (76–976)	150 (90–236)	0.438
Hematopoietic cell transplantation‐specific comorbidity index			0.600
0–1	36 (75.0)	28 (70.0)	
≥ 2	12 (25.0)	12 (30.0)	

### 3.2 Engraftment and Chimerism

Patients with high‐risk ALL who underwent either the Bu/Vp16/Cy/Flu/Ara‐C or Bu/Vp16/Cy conditioning regimen successfully achieved hematopoietic stem cell engraftment. Neutrophil and platelet engraftment times did not significantly differ between the groups (*p* = 0.506). The median time to neutrophil engraftment in the Bu/Vp16/Cy/Flu/Ara‐C group was 13 days, while platelet engraftment took 12 days. In contrast, the Bu/Vp16/Cy group experienced neutrophil engraftment in 11 days and platelet engraftment in 13 days. Complete donor chimerism was achieved in all patients within 1 month after transplantation.

### 3.3 Regimen‐Related Toxicity

The RRTs profiles of the two conditioning regimens are shown in Table 2. Oral mucositis and gastrointestinal toxicity were frequent adverse events in both high‐risk ALL patient groups post‐transplantation. Among them, two patients who underwent haploidentical transplantation developed severe oral mucositis, which necessitated a dose adjustment of MTX. Instead of the standard four doses (+1, +3, +6, +11 day), these patients received only three doses (+1, +3, +6 day). Hepatic, bladder, renal, central nervous system, and pulmonary toxicities occurred less frequently; the differences were not remarkable between the two groups. Notably, the Bu/Vp16/Cy group demonstrated significantly higher cardiac toxicity incidence (22.9% vs. 7.5%, *p* = 0.049), which may be attributed to high‐dose cyclophosphamide exposure. In the Bu/Cy/Vp‐16 regimen group, Grade I cardiac toxicity was most frequently observed (6 cases), manifesting as mild electrocardiographic abnormalities including nonspecific ST‐T changes in 3 cases, sinus arrhythmia in 2 cases, and occasional premature beats in 1 case. Grade II toxicity was documented in 4 cases, comprising moderate ST‐segment depression in 2 cases, supraventricular tachycardia in 1 case, and frequent atrial premature beats in 1 case. One patient developed Grade III cardiac toxicity, characterized by progressive reduction in QRS complex voltage exceeding 50% accompanied by pericardial effusion. In the Bu/Vp16/Cy/Flu/Ara‐C regimen group, there were 2 cases of Grade I cardiac toxicity (1 with nonspecific ST‐T changes and 1 with sinus arrhythmia) and 1 case of Grade II toxicity (one case of moderate ST‐segment depression).

**TABLE 2:** The regimen‐related toxicities of ALL patients who received transplantation.

	Bu/Vp16/Cy (*n* = 48)					Bu/Vp16/Cy/Flu/Ara‐C (*n* = 40)					*p*
	Total	Grade 1	Grade 2	Grade 3	Grade4	Total	Grade 1	Grade 2	Grade 3	Grade 4	
Oral mucositis	20 (41.7)	12 (25.0)	5 (10.4)	3 (6.3)	0 (0.0)	14 (35.0)	9 (22.5)	3 (7.5)	2 (5.0)	0 (0.0)	0.523
Gastrointestinal toxicity	19 (39.6)	10 (20.8)	6 (12.5)	3 (6.3)	0 (0.0)	15 (37.5)	7 (17.5)	5 (12.5)	3 (7.5)	0 (0.0)	0.842
Hepatic toxicity	4 (8.3)	2 (4.2)	2 (4.2)	0 (0.0)	0 (0.0)	3 (7.5)	2 (5.0)	1 (2.5)	0 (0.0)	0 (0.0)	0.886
Bladder toxicity	3 (6.3)	2 (4.2)	1 (2.1)	0 (0.0)	0 (0.0)	2 (5.0)	1 (2.5)	1 (2.5)	0 (0.0)	0 (0.0)	1.000
Cardiac toxicity	11 (22.9)	6 (12.5)	4 (8.3)	1 (2.1)	0 (0.0)	3 (7.5)	2 (5.0)	1 (2.5)	0 (0.0)	0 (0.0)	0.049
Renal toxicity	3 (6.3)	2 (4.2)	1 (2.1)	0 (0.0)	0 (0.0)	2 (5.0)	1 (2.5)	1 (2.5)	0 (0.0)	0 (0.0)	1.000
Central nervous system toxicity	2 (4.2)	1 (2.1)	1 (2.1)	0 (0.0)	0 (0.0)	2 (5.0)	1 (2.5)	1 (2.5)	0 (0.0)	0 (0.0)	1.000
Pulmonary toxicity	2 (4.2)	1 (2.1)	1 (2.1)	0 (0.0)	0 (0.0)	1 (2.5)	1 (2.5)	0 (0.0)	0 (0.0)	0 (0.0)	1.000

*Note:* Data are no. of patients (%).

### 3.4 GVHD

**FIGURE 1:**
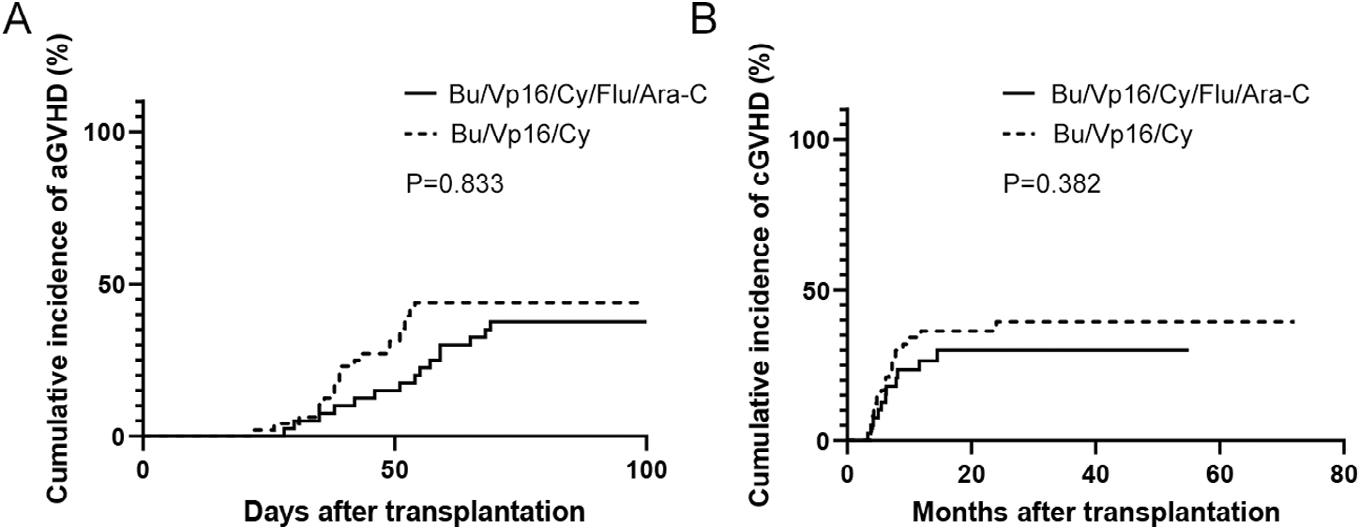
The cumulative incidence of acute and chronic GVHD in high‐risk ALL patients undergoing allo‐HSCT with either the Bu/Vp16/Cy/Flu/Ara‐C or Bu/Vp16/Cy preconditioning regimen. The rate of aGVHD (A) and cGVHD (B) in two cohorts.

GVHD following allo‐HSCT is a major element affecting patient prognosis and quality of life. We assessed the GVHD incidence in high‐risk ALL patients undergoing either the Bu/Vp16/Cy/Flu/Ara‐C or Bu/Vp16/Cy conditioning regimen. Analysis revealed no statistically significant increase in the risk of acute GVHD between groups (HR: 1.36, 95% CI: 0.71–2.61; *p* = 0.833). The cumulative incidences of acute GVHD were 43.8% (27.8%–56.2%) and 37.5% (20.5%–50.8%), respectively (Figure 1A). Incidence of grade I‐II aGVHD was 27.5% and 29.2% (*p* = 0.863), and grade III‐IV aGVHD rates were 10.0% and 14.6% (*p* = 0.517). No significant difference was observed in the 2‐year cumulative incidence of chronic GVHD between the groups (HR: 1.39, 95% CI: 0.67–2.89; *p* = 0.382). The incidence rates were 30.0% (13.2%–43.5%) and 39.5% (23.2%–52.3%), respectively (Figure 1B) or its subtypes (limited: 20.0% vs. 27.1%, *p* = 0.438; extensive: 7.5% vs. 10.4%, *p* = 0.636).

### 3.5 Relapse

**FIGURE 2:**
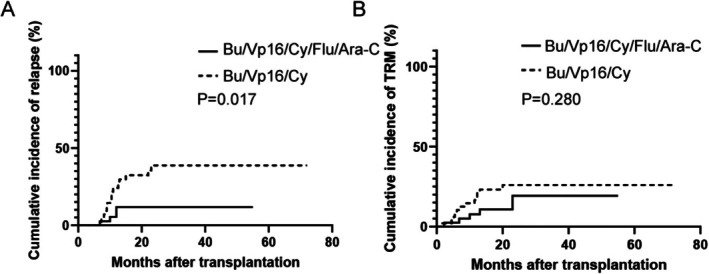
Relapse and TRM in high‐risk ALL Patients Undergoing Transplantation. The incidence of relapse (A) and TRM (B) is shown for patients receiving the Bu/Vp16/Cy/Flu/Ara‐C and Bu/Vp16/Cy regimens.

The Bu/Vp16/Cy/Flu/Ara‐C regimen significantly reduced the 2‐year relapse incidence compared with the Bu/Vp16/Cy regimen (11.8% (0.2% to 22.1%) vs. 38.7% (20.9% to 52.5%), *p* = 0.017) (Figure 2A). Among 19 patients experienced relapses, one discontinued further treatment, 10 received chemotherapy, and the remaining eight underwent immunotherapy (Bu/Vp16/Cy/Flu/Ara‐C group: 5 patients; Bu/Vp16/Cy group: 3 patients). Specific immunotherapies included four cases of CAR‐T cell therapy, two cases of TKI combined with donor lymphocyte infusion (DLI), and two cases of blinatumomab combined with DLI. By the end of the follow‐up period, four patients in the Bu/Vp16/Cy/Flu/Ara‐C group and one in the Bu/Vp16/Cy group survived after recurrence. Among the relapsed patients who received immunotherapy, two in the Bu/Vp16/Cy/Flu/Ara‐C cohort and one in the Bu/Vp16/Cy cohort were alive by the end of follow‐up.

### 3.6 Transplant‐Related Mortality

In addition to mortality from disease relapse, transplant‐related death significantly impacts the reduced survival rates of post‐transplant patients. The study found no significant difference in TRM between the Bu/Vp16/Cy/Flu/Ara‐C and Bu/Vp16/Cy groups. The cumulative incidences were 19.3% (3.5%–32.4%) and 26.0% (12.0%–37.7%), respectively, with a corresponding HR of 1.70 (0.67–4.30) (*p* = 0.28) (Figure 2B). The specific causes of TRM were as follows: in the Bu/Vp16/Cy group, 4 patients died from aGVHD, 3 from cGVHD, 3 from severe infections, and 2 from hemorrhagic cystitis. The Bu/Vp16/Cy/Flu/Ara‐C group experienced TRM due to aGVHD (2 patients), cGVHD (1 patient), severe infections (2 patients), and thrombotic microangiopathy (1 patient) (Table 3).

**TABLE 3:** The outcomes of ALL patients who underwent allo‐HSCT.

	Bu/Vp16/Cy (*n* = 48)	Bu/Vp16/Cy/Flu/Ara‐C (*n* = 40)	*p*
Relapse	15 (31.3)	4 (10.0)	0.016
aGVHD	21 (43.8)	15 (37.5)	0.553
I–II	14 (29.2)	11 (27.5)	
III–IV	7 (14.6)	4 (10.0)	
cGVHD	18 (37.5)	11 (27.5)	0.320
Limited	13 (27.1)	8 (20.0)	
Extensive	5 (10.4)	3 (7.5)	
Number of death	23 (48.0)	9 (22.5)	0.014
Cause of death			
Relapse	11 (23.0)	3 (7.5)	0.049
TRM	12 (25.0)	6 (15.0)	0.247
aGVHD	4 (8.3)	2 (5.0)	
cGVHD	3 (6.3)	1 (2.5)	
Infection	3 (6.3)	2 (5.0)	
Others	2 (4.2)	1 (2.5)	
Median follow‐up months	23.0 (1.2–72)	25.15 (2.3–55)	0.383

### 3.7 Overall Survival and Disease‐Free Survival

**FIGURE 3:**
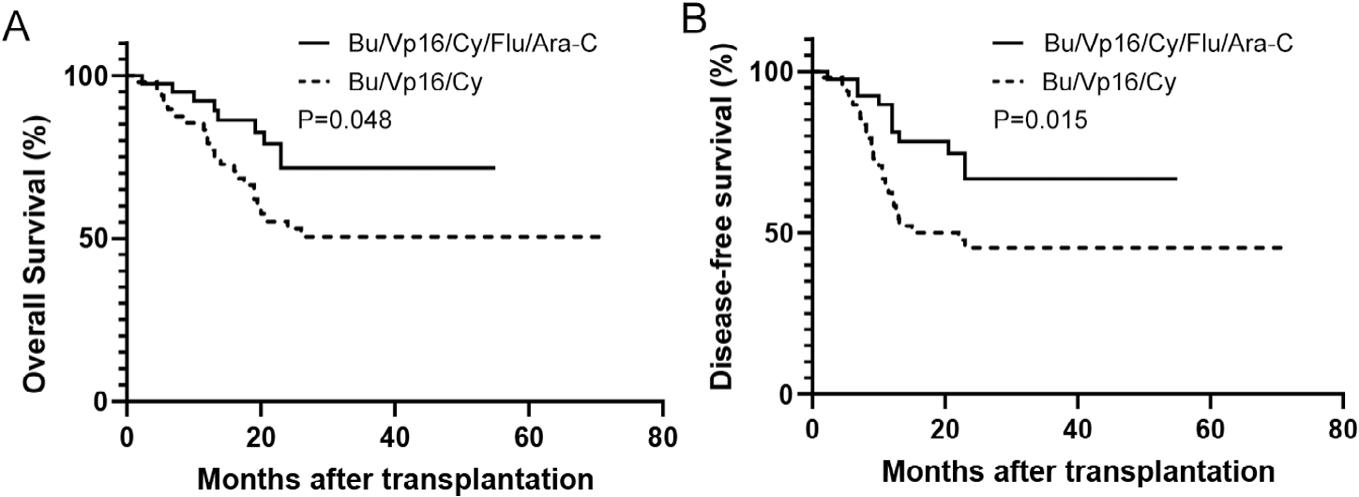
OS and DFS in high‐risk ALL Patients Undergoing allo‐HSCT. OS (A) and DFS (B) are presented for patients receiving the Bu/Vp16/Cy/Flu/Ara‐C and Bu/Vp16/Cy conditioning regimens.

Patients treated with the Bu/Vp16/Cy/Flu/Ara‐C regimen showed significantly better 2‐year OS at 71.6% (57.1% to 89.6%) compared to 50.6% (38%–67.3%) (*p* = 0.048), and improved two‐year DFS at 66.7% (51.9%–85.6%) versus 45.3% (33.1%–62.1%) (*p* = 0.015), relative to the Bu/Vp16/Cy group (Figure 3A,B).

### 3.8 Subgroup Analysis

**FIGURE 4:**
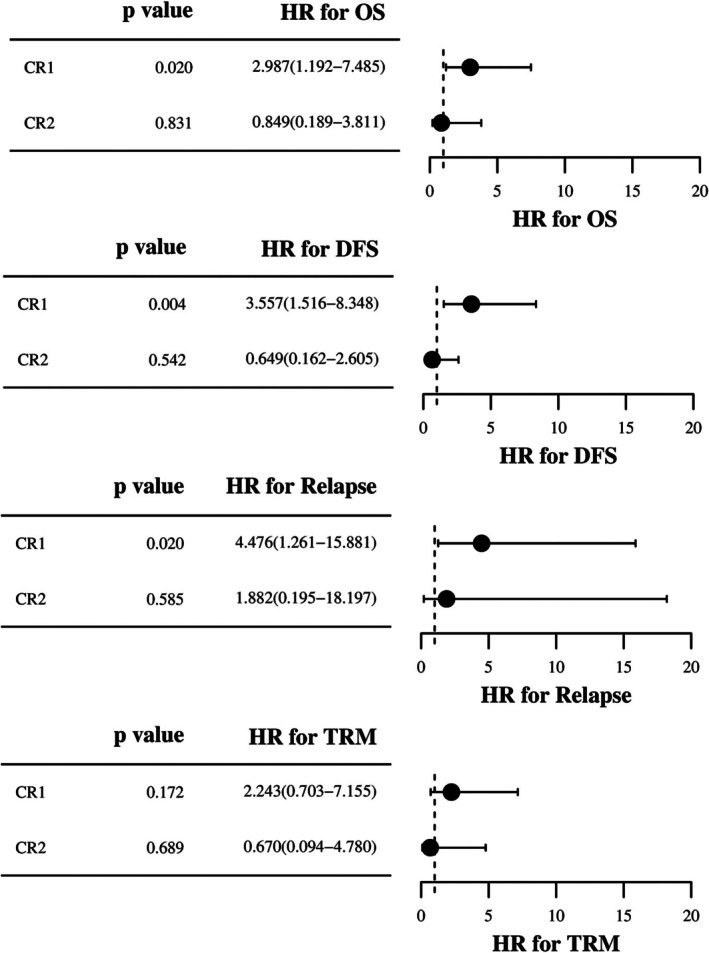
Subgroup analysis of OS, DFS, relapse and TRM stratified by number of pre‐transplant complete remissions in high‐risk ALL recipients receiving Bu/Vp16/Cy/Flu/Ara‐C versus Bu/Vp16/Cy regimens.

**FIGURE 5:**
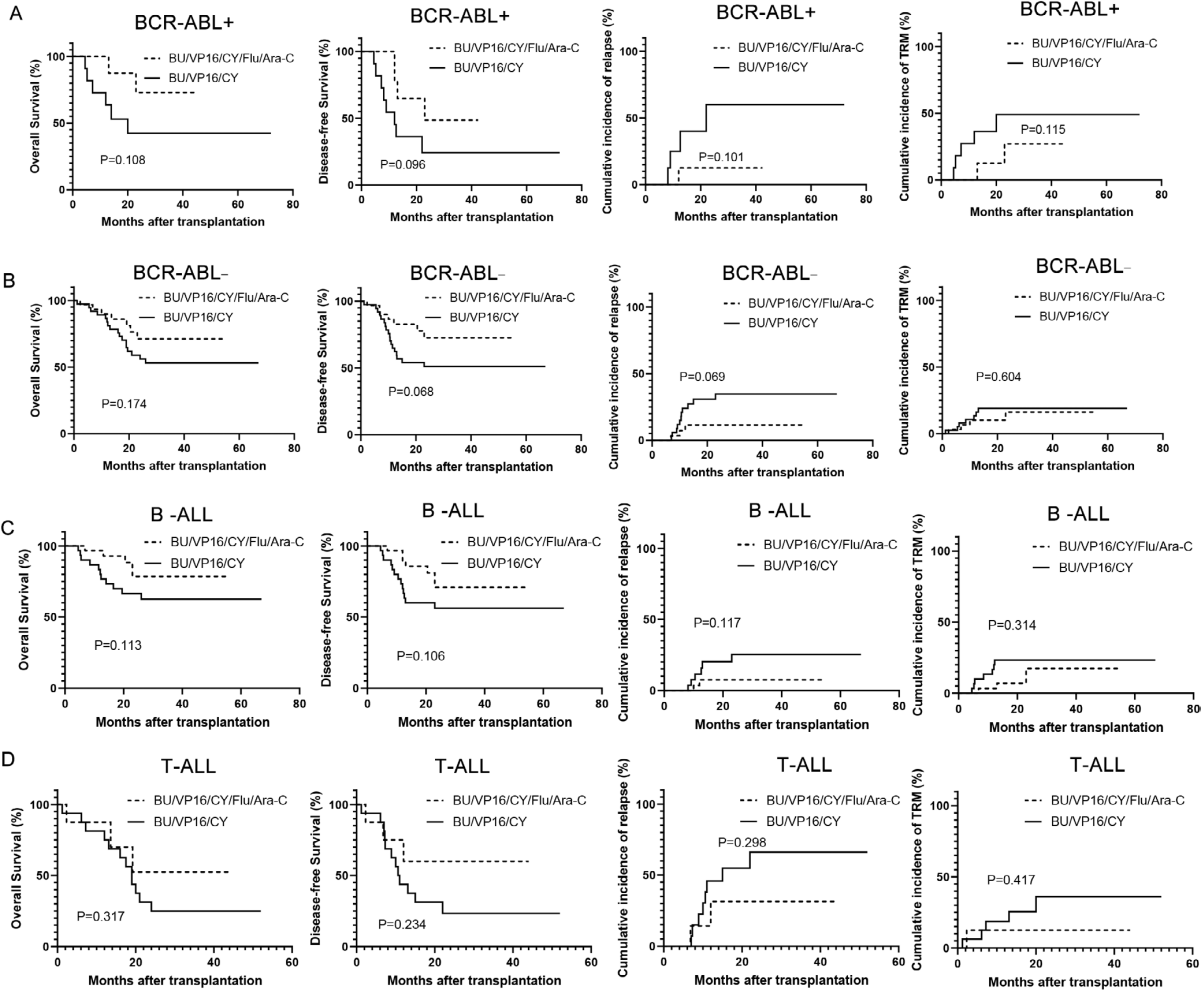
Subgroup Analysis of Relapse, TRM, OS, and DFS in high‐risk ALL patients stratified by Cytogenetic and Immunophenotypic Subtypes. BCR‐ABL^+^ and BCR:ABL^−^ (A, B); B‐ALL and T‐ALL (C, D).

Stratified analysis by disease status revealed that high‐risk CR1 patients undergoing Bu/Vp16/Cy/Flu/Ara‐C showed significant improvements in OS and DFS, with a notable decrease in cumulative relapse rates, while 2‐year TRM remained unchanged (Figure 4). In the ≥ CR2 subgroup, the two regimens demonstrated no statistically remarkable differences in two‐year OS, DFS, relapse rates and TRM (Figure 4). The Bu/Vp16/Cy/Flu/Ara‐C regimen improves OS and DFS in ALL patients, with significant benefits for high‐risk individuals in the CR1 group. In addition, we have performed additional subgroup analyses based on cytogenetic features and immunophenotype. The results demonstrated no statistically significant differences in outcomes between the two conditioning regimens, across all patient subgroups, including those defined by BCR‐ABL status (positive or negative) and immunophenotype (T‐cell or B‐cell). These data are presented in Figure 5A–D. It should be noted that 8 of 11 BCR::ABL‐positive patients (Bu/Cy/Vp‐16 group) and 7 of 10 (Flu/Ara‐C/Bu/Cy/Vp16 group) received TKI therapy.

### 3.9 Multivariate Cox Regression Analysis

We performed univariate and multivariate Cox regression analyses to determine independent risk factors affecting OS, DFS, and recurrence. Significant variables from the univariate analysis and variables known to have significant impacts on prognosis were included in the multivariate Cox regression model. The findings demonstrated that the conditioning regimen independently influenced OS, DFS, and relapse risk. Patients treated with the Bu/Vp16/Cy/Flu/Ara‐C preparative scheme demonstrated improved OS and DFS, as well as a significantly lower relapse risk compared with those in the Bu/Vp16/Cy group (Table 4). Pre‐transplant MRD‐positive patients had inferior OS and DFS compared to MRD‐negative patients (Table 4).

**TABLE 4:** Multivariable analysis for relapse, OS, and DFS in ALL patients underwent allo‐HSCT.

	Univariate analysis		Multivariate analysis	
	Hazard ratio (95% CI)	*p*	Hazard ratio (95% CI)	*p*
Relapse				
Patient age (≥ 35)	2.259 (0.658–7.753)	0.195	—	—
Gender (Male)	1.413 (0.516–3.589)	0.468		
Conditioning regimen (Bu/Vp16/Cy vs. Bu/Vp16/Cy/Flu/Ara‐C)	3.499 (1.161–10.544)	0.026	3.579 (1.191–10.863)	0.023
MRD+ vs. MRD—	1.882 (0.672–5.268)	0.229	1.957 (0.664–5.769)	0.224
aGVHD vs. no aGVHD	1.299 (0.522–3.232)	0.574	1.181 (0.442–3.153)	0.740
cGVHD vs. no cGVHD	1.017 (0.386–2.677)	0.973	0.918 (0.337–2.502)	0.867
OS				
Patient age (≥ 35)	1.609 (0.662–3.911)	0.294	—	—
Gender (Male)	1.374 (0.672–2.812)	0.384	—	—
Conditioning regimen (Bu/Vp16/Cy vs.Bu/Vp16/Cy/Flu/Ara‐C)	2.131 (1.059–4.286)	0.034	2.435 (1.159–5.118)	0.019
MRD+ vs. MRD—	3.411 (1.652–7.042)	0.001	3.336 (1.542–7.216)	0.002
aGVHD vs. no aGVHD	1.892 (0.858–4.171)	0.112	2.216 (0.979–4.616)	0.066
cGVHD vs. no cGVHD	1.563 (0.771–3.169)	0.215	1.520 (0.722–3.201)	0.270
DFS				
Patient age (≥ 35)	1.783 (0.783–4.061)	0.168	—	—
Gender (male)	1.365 (0.702–2.654)	0.359	—	—
Conditioning regimen (Bu/Vp16/Cy vs.Bu/Vp16/Cy/Flu/Ara‐C)	2.224 (1.098–4.502)	0.026	2.291 (1.128–4.652)	0.022
MRD+ vs. MRD—	2.455 (1.225–4.921)	0.011	2.335 (1.135–4.807)	0.021
aGVHD vs. no aGVHD	2.056 (1.076–3.926)	0.029	1.699 (0.858–3.365)	0.128
cGVHD vs. no cGVHD	1.522 (0.789–2.935)	0.210	1.290 (0.655–2.540)	0.461

## 4 Discussion

In this study, we further optimized the conditioning regimen based on the traditional Bu/Vp16/Cy regimen. Our findings demonstrate that, compared to the Bu/Vp16/Cy regimen, the Bu/Vp16/Cy/Flu/Ara‐C regimen significantly reduces the relapse rate and improves both OS and DFS in high‐risk ALL patients following transplantation. Importantly, this modified regimen also significantly reduces cardiotoxicity, maintains other RRTs within acceptable limits, and does not increase TRM.

Relapses remain one of the major challenges limiting the efficacy of transplantation in patients with ALL. Despite continuous improvements in transplantation techniques, the relapse rate after transplantation for ALL patients remains as high as 27.2%–47.5% [26, 27], posing a serious threat to long‐term survival. Given this urgent clinical need, the Chinese Society of Hematology has emphasized in relevant consensus documents the imperative to establish comprehensive and standardized strategies for the prevention and management of post‐transplant relapse [28]. Among the various factors influencing transplant outcomes, the choice of conditioning regimen is particularly critical. Currently, myeloablative conditioning based on either Bu‐Cy or TBI‐Cy represents the conventional standard [10]. To further enhance the anti‐leukemic effect and reduce relapse risk, this study explored the inclusion of Flu and Ara‐C to the traditional Bu/Vp16/Cy regimen, aiming to develop an optimized conditioning strategy.

Our results demonstrated that the Bu/Vp16/Cy/Flu/Ara‐C regimen significantly reduced the cumulative incidence of relapse compared to the conventional Bu/Vp16/Cy regimen (11.8% vs. 38.7%) in high‐risk ALL patients. Correspondingly, the 2‐year OS incidence was higher in the Bu/Vp16/Cy/Flu/Ara‐C cohort (71.6% vs. 50.6%). Recently, multiple studies have also been focusing on optimizing pretreatment regimens for ALL patients. One study reported a relapse rate of approximately 20% with a TBI‐Cy plus VP16 regimen, compared to 25% with TBI‐Cy alone [29]. Another study using an idarubicin‐intensified TBI‐Cy protocol showed a significantly lower relapse rate than conventional TBI‐Cy (19.1% vs. 40%), accompanied by improved 2‐year OS (66.2% vs. 45.3%) [19]. According to the results, the recurrence rate with our treatment regimen is not higher than that reported in other recent modified regimens. Furthermore, Tingting Cheng et al. recently modified the Bu‐Cy regimen by using a cladribine combined with intermediate‐dose Ara‐C intensified Bu/Cy (CBAC) regimen for high‐risk B‐ALL patients, with TBI‐Cy as the control. Their results showed a lower relapse rate in the CBAC group compared to the TBI‐Cy group (17.3% vs. 35.7%), with 2‐year OS rates of 74.3% and 66.8%, respectively [30]. In our subgroup analysis of B‐ALL patients, the Bu/Vp16/Cy/Flu/Ara‐C regimen was associated with a 2‐year relapse incidence of 7.15% and a 2‐year OS of 78.1%. The relapse rate in our study was lower than that observed with the CBAC regimen, while the OS was similar. This difference may be attributed to the relatively small sample size of this subgroup. Taken together, integrating existing literature with our data, the Bu/Vp16/Cy/Flu/Ara‐C regimen demonstrates promising application prospects in the conditioning for transplantation in high‐risk ALL patients.

In clinical decision‐making, the toxicity of the conditioning regimen is also a critical factor that must be carefully considered alongside reducing post‐transplant relapse risk. Physicians need to strike an optimal balance between effectively controlling disease recurrence and avoiding excessive RRTs and TRM. In the preparative regimen, Cy is one of the drugs contributing to RRTs. One study has shown that even within the conventional clinical dose range (120 or 100 mg/kg) in the conditioning regimen, Cy‐induced cardiac adverse events remain a significant concern [18]. To mitigate the toxicity burden of Cy, this study attempted to reduce its dose in the regimen. However, dose reduction may raise the risk of graft failure. To address this dilemma, we introduced fludarabine as a potent immunosuppressive agent, aiming to reduce toxicity while maintaining stable engraftment. We did not completely replace Cy with Flu, as previous studies have shown that the cumulative incidence of primary graft failure was significantly higher with the Bu‐Flu regimen compared to the Bu‐Cy regimen (17.6% vs. 3.0%, *p* = 0.04) [31].

In the combined Bu/Vp16/Cy/Flu/Ara‐C regimen used in this study, all ALL patients successfully achieved graft engraftment. Encouragingly, compared to the Bu/Vp16/Cy regimen, the new regimen significantly reduced the incidence of cardiotoxicity (7.5% vs. 22.9%). However, no significant differences were observed between the two groups in terms of other toxicities or TRM (TRM: 19.3% vs. 26%). Wei Sun et al. also supports the strategy of dose adjustment within the Bu‐Cy framework. In their Bu/Cy/Flu/Ara‐C regimen with Cy reduced to 1.0 g/m^2^/day for two consecutive days in patients with a Hematopoietic Cell Transplantation Comorbidity Index ≥ 3, all patients successfully achieved neutrophil engraftment. The two‐year cumulative TRM was 25.1%, and the cardiotoxicity incidence was 13.9% [32]. In contrast, a study using the Bu/Vp16/Cy regimen (Cy dose 120 mg/kg) in autologous transplantation for lymphoma reported a cardiotoxicity incidence of 11.5% [33]. Haiyan Zhang et al.'s [10] study on standard‐risk B‐ALL transplantation reported that the cardiotoxicity incidence was only 6.2% with either the Bu‐Cy or TBI‐Cy regimen using the standard Cy dose (120 mg/kg), which is lower than the 22.9% observed with the pre‐modified Bu/Vp16/Cy regimen in our study. This discrepancy may be related to differences in study population characteristics and specific treatment protocol details, particularly since our study specifically focused on high‐risk ALL patients, and potential variations may exist in the precise drug combinations or dose intensities. After protocol optimization, the Bu/Cy/Flu/Ara‐C regimen in our study controlled the cardiotoxicity incidence to 7.5%. In summary, our regimen demonstrates that appropriately reducing the Cy dose while combining it with Flu can effectively lower the risk of cardiotoxicity while ensuring stable engraftment, providing valuable clinical insights for optimizing conditioning strategies in high‐risk ALL patients.

Although the modified regimen employed in this study significantly improved outcomes for ALL patients undergoing transplantation, we acknowledge several limitations. First, the retrospective design may introduce selection bias and the influence of confounding factors; therefore, the efficacy and safety of this regimen still require validation through prospective randomized controlled trials. Second, the study did not conduct power analysis. The current sample size may be relatively small, potentially leading to insufficient statistical power and affecting the robustness of the findings. Future research should incorporate scientific power calculations during the design phase and further validate the findings of this study in larger, independent cohorts to enhance reliability and generalizability.

In summary, the Bu/Vp16/Cy/Flu/Ara‐C preparative protocol effectively reduces the risk of relapse after transplantation in high‐risk ALL patients and significantly improves OS and DFS, with particularly notable benefits for patients in CR1. Furthermore, the regimen demonstrates a favorable safety profile, not only significantly reducing the risk of cardiotoxicity but also without substantially increasing other RRTs or TRM. Therefore, Bu/Vp16/Cy/Flu/Ara‐C can be considered an effective and safe conditioning strategy for allo‐HSCT in high‐risk ALL patients.

## Author Contributions

**Xiaoyan Zhao:** writing – original draft, conceptualization. **Yifan Yao:** formal analysis, data curation. **Yan Sun:** formal analysis, investigation. **Ziwei Xu:** software. **Aiguo Liu:** resources, validation. **Xin Dong:** visualization, writing – review and editing. **Huafang Wang:** supervision, funding acquisition, writing – review and editing.

## Ethics Statement

The Ethics Committee of Union Hospital, Huazhong University of Science and Technology approved the study protocol. Written informed consent was obtained from all participants. This study adhered to the Declaration of Helsinki principles.

## Conflicts of Interest

The authors declare no conflicts of interest.

## Data Availability

The data supporting this study are available from the corresponding author upon reasonable request.
